# Efficacy and safety of immunotherapy combined with single-agent chemotherapy as second- or later-line therapy for metastatic non-small cell lung cancer

**DOI:** 10.3389/fimmu.2023.1086479

**Published:** 2023-09-18

**Authors:** Dongna Chen, Lin Li, Mingzhao Wang, Xingsheng Hu, Jun Jiang, Weihua Li, Lin Yang, Meng Fan, Yuankai Shi, Fang Lv, Yutao Liu

**Affiliations:** ^1^ Department of Medical Oncology, Beijing Chao Yang District San Huan Cancer Hospital, Beijing, China; ^2^ Department of Diagnostic Radiology, National Cancer Center/National Clinical Research Center for Cancer/Cancer Hospital, Chinese Academy of Medical Sciences and Peking Union Medical College, Beijing, China; ^3^ Department of Medical Oncology, National Cancer Center/National Clinical Research Center for Cancer/Cancer Hospital, Chinese Academy of Medical Sciences and Peking Union Medical College, Beijing, China; ^4^ Department of Diagnostic Image, National Cancer Center/National Clinical Research Center for Cancer/Cancer Hospital, Chinese Academy of Medical Sciences and Peking Union Medical College, Beijing, China; ^5^ Department of Pathology, National Cancer Center/National Clinical Research Center for Cancer/Cancer Hospital, Chinese Academy of Medical Sciences and Peking Union Medical College, Beijing, China; ^6^ Research and Development Department, EVbio Technology Co., Ltd., Beijing, China; ^7^ Department of Thoracic Surgery, National Cancer Center/National Clinical Research Center for Cancer/Cancer Hospital, Chinese Academy of Medical Sciences and Peking Union Medical College, Beijing, China

**Keywords:** non-small cell lung cancer, immunotherapy, chemotherapy, EV markers, EGFR, VEGFR2

## Abstract

**Objective:**

This study sought to assess the efficacy and safety of immunotherapy combined with single-agent chemotherapy as a second- or later-line setting for metastatic non-small cell lung cancer (NSCLC) and to provide clinical evidence for this treatment regimen. The predictive value of extracellular vesicle (EV) membrane proteins was explored in patients who underwent this treatment.

**Methods:**

Clinical data from patients diagnosed with metastatic NSCLC who received immunotherapy plus single-agent chemotherapy as a second- or later-line setting were retrospectively collected between March 2019 and January 2022. A total of 30 patients met the inclusion criteria, and all were pathologically confirmed to have NSCLC. Short-term efficacy, progression-free survival (PFS), EV markers for response prediction, and adverse events were assessed.

**Results:**

Efficacy data were available for all 30 patients and included a partial response in 5 patients, stable disease in 18 patients, and disease progression in 7 patients. The objective response rate was 16.7%, the disease control rate was 76.7%, and the median PFS was 3.2 months. Univariate analysis showed that PFS was not associated with sex, age, smoking status, treatment lines, prior use of immunotherapy, or prior use of antiangiogenic drugs. The EV membrane proteins MET proto-oncogene, receptor tyrosine kinase (c-MET), epidermal growth factor receptor (EGFR), and vascular endothelial growth factor receptor 2 (VEGFR2) at baseline were associated with poor prognosis and correlated with the efficacy of immunotherapy plus chemotherapy. According to the receiver operating characteristics and Kaplan–Meier curve analyses, patients with high c-MET, EGFR, and VEGFR2 expression at baseline had significantly shorter PFS than those with low expression. In addition, VEGFR2 expression was increased after combined immunotherapy in responders, which was decreased in non-responders. The most common grade 2 or higher adverse events were neutropenia, gastrointestinal reactions, and thyroid dysfunction, all of which were tolerated.

**Conclusions:**

Immunotherapy plus single-agent chemotherapy as a second- or later-line treatment is safe, effective, and tolerable for metastatic NSCLC. EV markers can be used as predictive markers of efficacy in patients with metastatic NSCLC treated with immunotherapy plus chemotherapy to help monitor treatment efficacy and guide treatment decisions.

## 1 Introduction

Lung cancer is one of the most common malignancies, and it has the highest mortality rate of all cancer types in China and worldwide. There are 733,000 newly diagnosed lung cancer cases and 610,000 deaths each year in China (1). Non-small cell lung cancer (NSCLC) accounts for approximately 85% of lung cancer cases. At the time of diagnosis, up to 60% of patients have locally advanced or metastatic NSCLC, with a 5-year survival rate of approximately 5% (2). Although genetic testing can identify patients with therapeutically targeted alterations including epidermal growth factor receptor (*EGFR*)-sensitive mutations and anaplastic lymphoma kinase (*ALK*) gene rearrangements who may be treated with targeted therapies, most patients experience disease progression within 12-18 months.

The conventional chemotherapy (docetaxel and pemetrexed) is the current standard of care for the second-line treatment of patients who experience disease progression after first-line therapy (3). Shepherd et al. (4) and Fossella et al. (5) reported that the objective response rate (ORR) of patients with NSCLC treated with docetaxel is approximately 7% NSCLC. The phase 3 clinical study reported by Hanna et al. (6) in 2004 found that the ORR of patients with stage III/IV NSCLC treated with pemetrexed as the second-line therapy was 9.1%. Immune checkpoint inhibitors have emerged as a new treatment regimen for the second-line treatment of both squamous and non-squamous NSCLC. The Checkmate078 (7) study found that the use of nivolumab as a second-line treatment for Chinese patients with NSCLC significantly prolonged overall survival (OS) (median: 12.0 vs. 9.6 months, *p* = 0.0006) and increased ORR (16.6% vs. 4.2%, *p* < 0.0001) with limited adverse events. The National Medical Products Administration (NMPA) of China approved the indication for nivolumab as a second-line therapy in 2018. However, evidence supporting the use of immunotherapy combined with chemotherapy as a second-line treatment for advanced NSCLC therapies is lacking.

Extracellular vesicle (EVs) are lipid bilayer membrane-bound vesicles secreted into the circulation by almost all cell types. EVs are widely distributed in diverse body fluids, including plasma, whole blood, urine, saliva, cerebrospinal fluid, and breast milk (8). Previous studies have shown that that EVs emerged as a diagnostic tool for early cancer detection, disease monitoring, and treatment evaluation (9–11). However, the potential clinical utility of plasma EV surface membrane proteins in advanced NSCLC patients who received immunotherapy remains elusive.

Thus, the purpose of this study was to evaluate the efficacy and safety of immunotherapy combined with single-agent chemotherapy as a second- or later-line treatment for metastatic NSCLC and to provide clinical evidence for the use of the regimen. We also aimed to explore the clinical application value of EV surface membrane proteins in metastatic NSCLC patients who received immunotherapy combined with single-agent chemotherapy as a second- or later-line therapy.

## 2 Materials and methods

### 2.1 General data

Clinical data for patients with advanced NSCLC who received immunotherapy combined with single-agent chemotherapy as a second- or later-line therapy in the Cancer Hospital of the Chinese Academy of Medical Sciences and the Beijing Chao yang District San huan Cancer Hospital between March 2019 and January 2022 were collected. The inclusion criteria were a) patients who had histologically or cytologically confirmed metastatic or recurrent (stage IV) NSCLC, (b) patients who had received platinum-based regimens or targeted therapy but had relapsed or not responded to the treatment, c) patients who had received immunotherapy combined with single-agent chemotherapy as a second- or later-line therapy, d) patients with at least one measurable tumor lesion diagnosed by computed tomography (CT) or magnetic resonance imaging (MRI) with a maximum diameter of ≥10 mm, as measured by spiral CT or MRI, and e) patients with complete clinical and survival data. The exclusion criteria were a) patients with an indefinite cytological or pathological diagnosis, b) patients with a history of non-infectious pneumonia requiring glucocorticoid therapy within 1 year prior to the first drug administration, and c) patients with symptomatic central nervous system metastases. Based on the inclusion and exclusion criteria, 30 patients were eligible for this study. This study was approved by the Ethics Committee of the Cancer Hospital of the Chinese Academy of Medical Sciences (approval Number: 20/361-2145). All patients signed a written informed consent to participate before the study in accordance with the Declaration of Helsinki.

### 2.2 Treatment

All patients received programmed cell death-1 (PD-1) inhibitors (pembrolizumab, sintilimab, camrelizumab, and nivolumab in 14, 11, 3 and 2 patients, respectively) combined with single-agent chemotherapy (albumin-bound paclitaxel, paclitaxel liposomes, vinorelbine soft capsules, pemetrexed, and gemcitabine in 15, 6, 5, 3, and 1 patients, respectively) (**Table S1**). The regimen was prescribed by the investigator after evaluation. Pretreatment was performed according to the instructions of the prescribed drug. Efficacy was evaluated after two cycles of treatment lasting 21 days. Each patient received routine blood, biochemistry, thyroid function, myocardial enzyme testing, and electrocardiogram before each cycle.

### 2.3 Evaluations and criteria

The ORR, progression-free survival (PFS), EV marker levels for response prediction, and adverse events were evaluated in all patients. Imaging results were collected as baseline data within 1 month prior to treatment, and radiographic examinations were performed after every two cycles of immunotherapy combined with single-agent chemotherapy to assess drug efficacy. The efficacy evaluation was performed using the Response Evaluation Criteria in Solid Tumors (RECIST) Version 1.1 (12), which grades the response as a complete response (CR), partial response (PR), stable disease (SD), or progressive disease (PD). Responders were defined as patients who benefited from the treatment with PR or SD. Non-responders were defined as patients who did not benefit from the treatment with PD. ORR was defined as CR or PR. Disease control rate (DCR) was defined as CR, PR, or SD. PFS was defined as the time from relapse or non-response after 1–2 courses of prior systemic treatment to objective disease progression or death from any cause. Peripheral whole-blood specimens (10 ml per sample) were collected through venipuncture into EDTA tubes and centrifuged at 4°C and 3000×g for 15 min. Isolated plasma, serum, lymphocytes, and peripheral blood monocular cells (PBMCs) were placed at −80°C for long-term storage. Clinical data for patients, including baseline characteristics, treatment regimens, laboratory testing results, pathologic examination results, and CT images, were acquired with individual medical records. The severity of adverse events was evaluated using the Common Terminology Criteria for Adverse Events (CTCAE) 4.0. Follow-up ended on January 21^st^, 2022.

### 2.4 Specimen preparation

Precise 10 ml peripheral blood prior to treatment (at baseline, stage D1) and after two cycles of immunotherapy plus single-agent chemotherapy (stage D2) was collected with an EDTA tube, centrifugated at 4°C, 3000× g, and 10 min for separating plasma and blood cells. Plasma samples were performed for EV expression array analysis.

### 2.5 EV expression array analysis

Antibodies were diluted to 200 μg/ml with 5% glycerol and then printed onto a 3D modified slide surface (Capital Biochip Corp, Beijing, China) using an Arrayjet microarrayer (Roslin, UK). A total of 57 antihuman antibodies were used for capturing EV proteins (**Table S2**). PBS with 5% glycerol and 10 μg/ml of biotinylated bovine serum albumin (BSA) were used as negative and positive controls, respectively. The EV expression array assay was performed by referring to previous studies (13, 14). Briefly, the microarray slides were incubated with a 10 μl unpurified plasma sample diluted (1:10) in wash buffer (0.05% Tween20 in PBS) at room temperature for 2 h followed by overnight incubation at 4°C. After a wash, the slides were incubated with biotinylated detection antibodies (anti-human-CD9, -CD63 and -CD81, LifeSpan BioSciences, WA, USA) diluted 1:1500 in wash buffer. After washing, the slides were incubated with Cy3-labeled streptavidin (Life Technologies) diluted 1:1500 in wash buffer for detection. The microarray slides were scanned using the GenePix 4000A microarray scanner (Molecular Devices, CA, USA). The signal intensity of the fluorescent images was extracted using the GenePix Pro image analysis software (Molecular Devices, CA, USA). All antibodies were printed in triplicate, and the mean value of the total signal was used to estimate the signal intensity.

The expression level of EV proteins at baseline in responders vs. non-responders was compared. EV proteins with *p* < 0.05 and fold change (FC) > 1.5 were significantly upregulated, and those with *p* < 0.05 and FC < 0.67 were significantly downregulated in responders.

### 2.6 Statistical analysis

Enumeration data are presented as counts and rates (%). Kaplan–Meier (KM) survival analysis was performed to describe the survival of the patients. Volcano plots were performed using the OmicStudio tools at https://www.omicstudio.cn/tool/7. Differences among the subgroups were analyzed by the Mann–Whitney test or t test. Graphs and statistics were performed using GraphPad Prism 6.0 or SPSS21.0. Receiver operating characteristic (ROC) curves were generated to derive the best cut-offs. The area under the ROC curve (AUC) refers to the area between the ROC curve and the x-axis. *p* < 0.05 (*) indicates statistical significance.

## 3 Results

### 3.1 Baseline characteristics of patients

A total of 30 metastatic NSCLC patients receiving immunotherapy combined with single-agent chemotherapy as a second- or later-line therapy were enrolled in this study. The clinical data at baseline are shown in **Table 1**. The patients were 33–74 years old [median: 59 years; ≥ 65 years: 11 patients (36.7%), < 65 years: 19 patients (63.3%)], and 23 were male (76.7%). In all, 20 patients with lung adenocarcinoma and 10 patients with lung squamous cell carcinoma were included, and 2 (6.7%) and 28 (93.3%) patients presented with Eastern Cooperative Oncology Group Performance Status (ECOG PS) of 0 and 1, respectively. Metastases to the bone, brain, adrenal gland, liver, spleen, and kidney occurred in 15 (50.0%), 10 (33.3%), 7 (23.3%), 4 (13.3%), 2 (6.7%), and 1 (3.3%) patient(s), respectively. Of the 25 patients who underwent actionable alteration detection, 4 harbored *EGFR* L858R, 4 harbored *EGFR* 19del, 3 harbored *EGFR* T790M, and 3 harbored *BRAF* V600E; each patient harbored *KRAS* mutation, *HER2* amplification, *HER2* 20ins, *ROS1* rearrangement, *ALK* fusion, and *RET* rearrangement; 4 harbored *TP53* alterations, and 7 were negative for actionable alterations. In addition, 4 patients harboring actionable alterations at initial diagnosis who were then resistant to TKIs were included among the 25 eligible patients, such as 1 *EGFR* 19del-positive patient who acquired *TP53* alteration and *ROS1* rearrangement from EGFR-TKI, 1 *EGFR* 19del-positive patient who acquired *TP53* alteration and *EGFR* T790M from EGFR-TKI, 1 *EGFR* 19del-positive patient who acquired *EGFR* T790M from EGFR-TKI, and 1 *EGFR* L858R-positive patient who acquired *EGFR* T790M from EGFR-TKI. A total of 8 patients (26.7%) underwent the programmed cell death ligand-1 (PD-L1) detection with a median TPS of 80% (range: 2%-95%), and the PD-L1 status of the remaining 22 patients (73.3%) was unknown. Overall, 19 patients were smokers (63.3%), 17 (56.7%) had received second-line therapy, and 13 (43.3%) had received third-line therapy; 16 patients (53.3%) were previously treated with immunotherapy, and 21 (70%) had prior use of antiangiogenic therapy.

**Table 1:** Baseline clinical data of included NSCLC patients.

	Characteristic	Cases (n, %)
**Sex**	Male	23 (76.7)
	Female	7 (23.3)
**Age**	≥65 years	11 (36.7)
	<65 years	19 (63.3)
**Pathological subtype**	LUAD	20 (66.7)
	LUSC	10 (33.3)
**Smoking history**	Yes	19 (63.3)
	No	11 (36.7)
**ECOG PS**	0	2 (6.7)
	1	28 (93.3)
**Metastases**	Bone	15 (50.0)
	Brain	10 (33.3)
	Adrenal gland	7 (23.3)
	Liver	4 (13.3)
	Spleen	2 (6.7)
	Kidney	1 (3.3)
**Actionable alteration status**	*EGFR* L858R	4 (13.3)
	*EGFR* 19del	4 (13.3)
	*EGFR* T790M	3 (10.0)
	*BRAF* V600E	3 (10.0)
	*KRAS* mutations	1 (3.3)
	*HER2* amplification	1 (3.3)
	*HER2* 20ins	1 (3.3)
	*ROS1* rearrangement	1 (3.3)
	*ALK* fusion	1 (3.3)
	*RET* rearrangement	1 (3.3)
	*TP53*alterations	4 (13.3)
	Without	7 (23.3)
	Unknown	5 (16.7)
**PD-L1 status**	TPS ≥ 1	6 (20.0)
	Unknown	24 (80.0)
**Line of treatment**	Second-line	13 (43.3)
	Third- or later-line	17 (56.7)
**Prior use of immunotherapy**	Yes	16 (53.3)
	No	14 (46.7)
**Prior use of antiangiogenic agents**	Yes	21 (70.0)
	No	9 (30.0)

NSCLC, non-small cell lung cancer; LUAD, lung adenocarcinoma; LUSC, lung squamous cell carcinoma; ECOG PS, Eastern Cooperative Oncology Group Performance Status; PD-L1, programmed cell death ligand-1; TPS, tumor proportion score; del, deletion; ins, insertion; without, patients who did not harbor actionable alterations; unknown, patients who were not performed on for actionable alteration detection.

### 3.2 Efficacy evaluation

Of the 30 enrolled patients, none achieved CR, 5 (16.7%) achieved PR, 18 (60.0%) achieved SD, and 7 (23.3%) experienced PD. The ORR and DCR were 16.7% and 76.7%, respectively. The median PFS was 3.2 months (95% confidence interval [CI]: 2.7–3.7, range: 1.0-10.6). The KM curves for PFS is described in **Figure S1**. Univariate analysis revealed that PFS was not associated with (*p* > 0.05) sex, age, brain metastasis status, ECGO PS, pathological subtype, smoking status, treatment lines, prior use of immunotherapy, or prior use of antiangiogenic therapy (**Table 2**).

**Table 2:** Univariate analysis of PFS.

Clinical characteristics	No. of cases	Median PFS (mo)	Log-rank *p* value
Sex			
Male	23	3.0	1.0
Female	7	2.8	1.0
Age (years)			
≥65	11	2.9	1.0
<65	19	3.0	1.0
Smoking history			
No	11	2.8	1.0
Yes	19	3.1	
Brain metastases			
With	10	3.0	0.943
Without	20	3.2	
ECOG PS			
0	2	1.5	0.321
1	28	3.5	
Pathological subtype			
LUAD	20	3.2	0.807
LUSC	10	2.9	
Line of treatment			
Second-line	13	2.9	0.698
Third- or later-line	17	3.0	0.698
Prior use of immunotherapy			
Yes	16	2.9	0.226
No	14	2.9	0.226
Prior use of antiangiogenic agents			
Yes	21	2.9	1.0
No	>9	3.8	1.0

ECOG PS, Eastern Cooperative Oncology Group Performance Status; LUAD, lung adenocarcinoma; LUSC, lung squamous cell carcinoma; No., number; mo, month; PFS, progression-free survival.

### 3.3 EV markers for response prediction

Among the 30 patients, 14 (3 PR, 7 SD, 4 PD) patients who had plasma samples prior to treatment were selected for EV Array analysis. This assay assesses EV membrane proteins related to the immune system, immune typing, chemoradiotherapy, vascular therapy (famitinib and anlotinib), and lung cancer, as well as the common EV protein markers CD63, CD81, and CD9 (15, 16). Then, these samples were analyzed to obtain the membrane protein expression profiles of the EVs. The results are detailed in the following subsections.

#### 3.3.1 Heatmap analysis of the protein expression clusters

**Figure 1:**
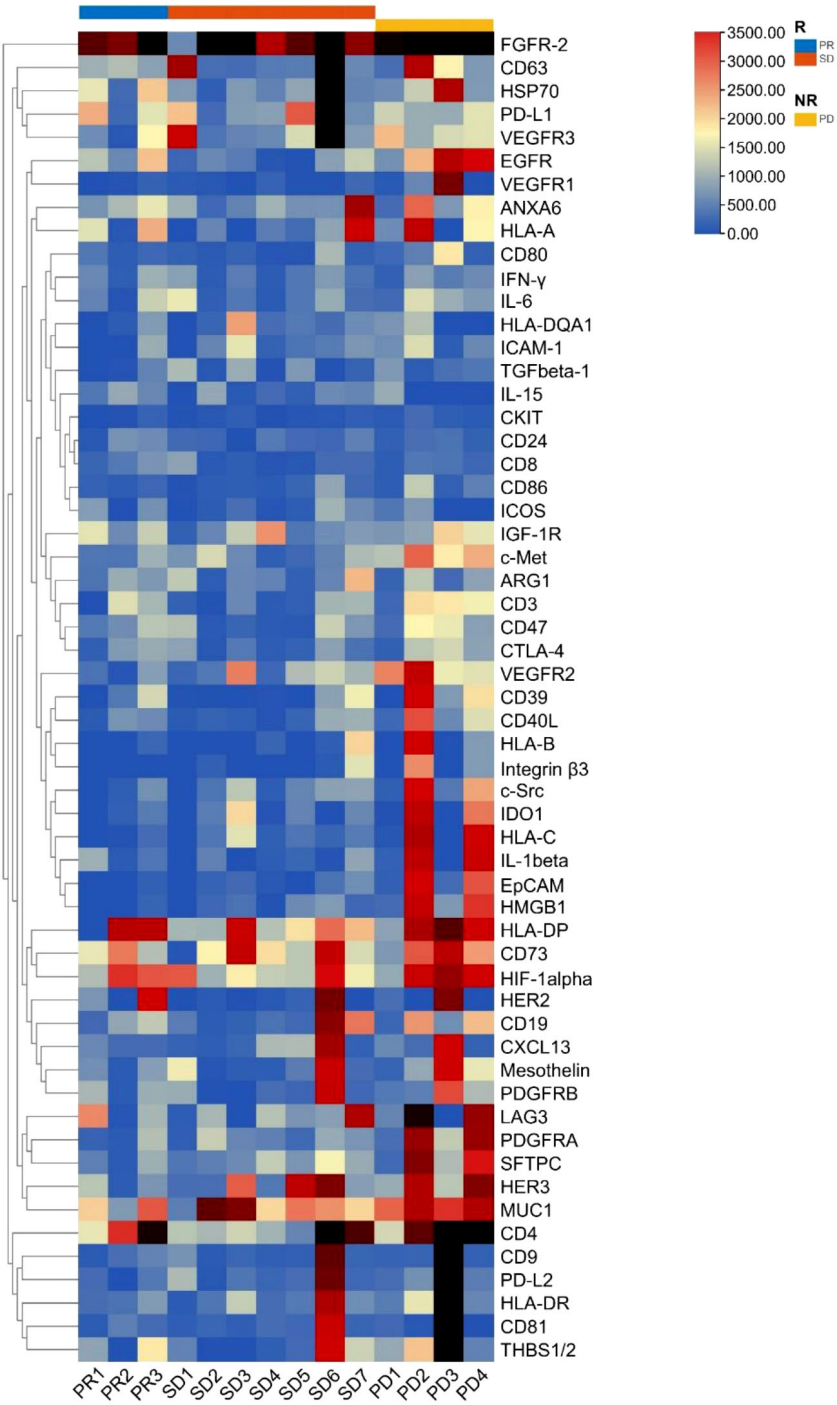
Cluster heatmap analysis of plasma EV membrane proteins at baseline in non-small cell lung cancer patients (n = 14). EV, extracellular vesicle; R, responders, NR, non-responders; PR, partial response; SD, stable disease; PD, progressive disease.

The cluster heatmap analysis of EV membrane proteins at baseline in patients who benefited from the treatment (responders: 3 PR and 7 SD) and those who showed no treatment benefit (non-responders: 4 PD) is depicted in **Figure 1**. We found that most plasma EV membrane proteins had higher expression levels in non-responders than responders.

#### 3.3.2 Volcano map analysis

**Figure 2:**
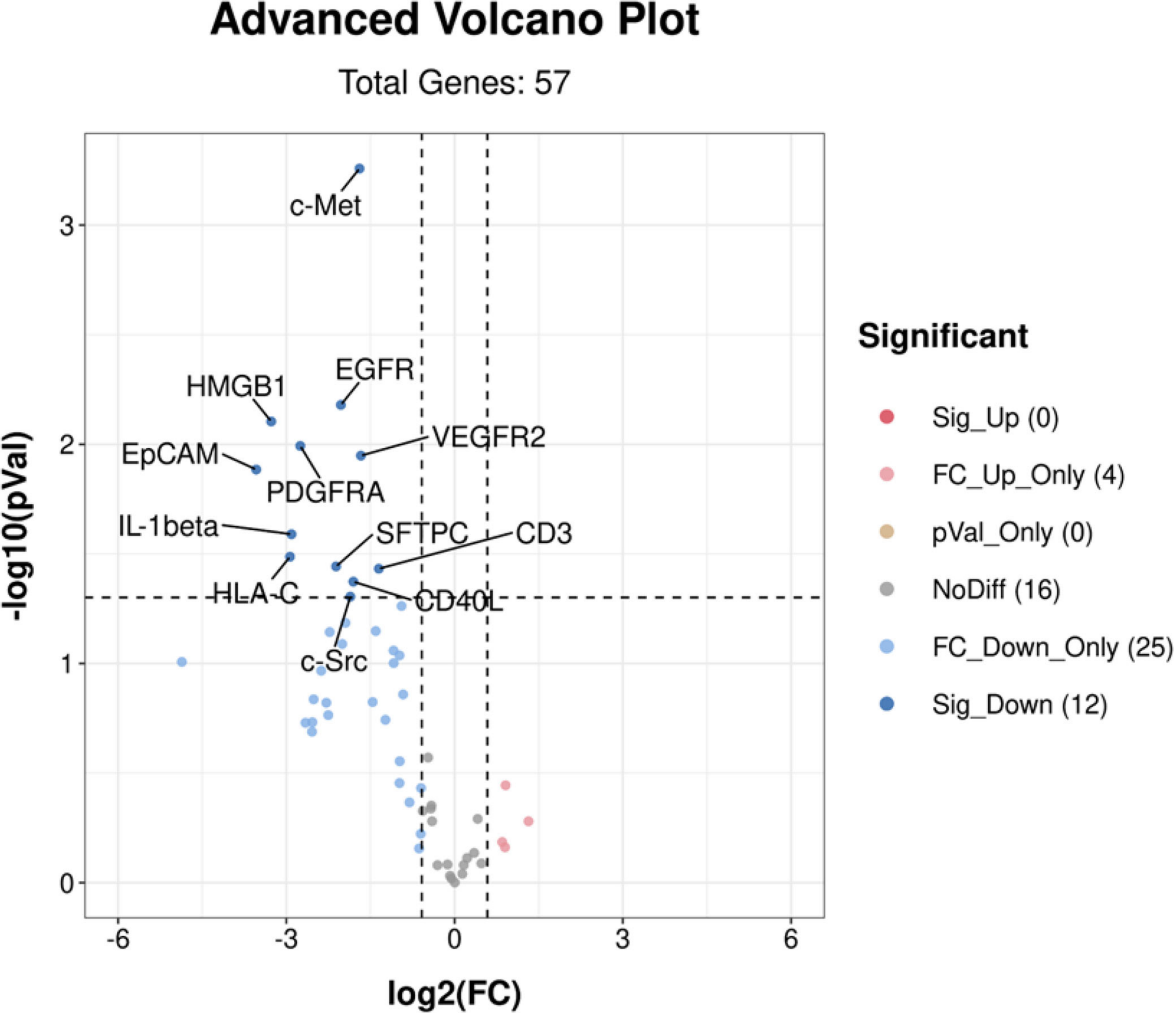
Volcano plot analysis of differentially expressed EV membrane proteins at baseline between responders (n = 10) and non-responders (n = 4). A total of 12 EV membrane proteins showed significantly lower expression in responders than non-responders. Sig_Up (0) indicated that no significantly upregulated EV proteins (*p* < 0.05 and FC > 1.5) were identified in responders; FC_Up_Only (4) indicated 4 EV proteins with *p* > 0.05 and FC > 1.5 identified in responders; pVal_Only (0) indicated that there were no EV proteins with *p* < 0.05 and 0.67 < FC < 1.5 identified in responders; NoDiff (16) indicated 16 EV proteins with *p* > 0.05 and 0.67 < FC < 1.5 identified in responders; FC_Down_Only (25) indicated 25 EV proteins with *p* > 0.05 and FC < 0.67 identified in responders; Sig-down (12) indicated 12 EV proteins with *p* < 0.05 and FC < 0.67 identified in responders. EV, extracellular vesicle; FC, fold change. *p*-values were calculated using the Mann–Whitney test.

Volcano plot analysis of the EV membrane proteins was performed for responders (3 PR and 7 SD) and non-responders (4 PD). The results are shown in **Figure 2**. In responders, 12 EV membrane proteins at baseline showed significantly lower expression (Mann–Whitney test, *p* < 0.05, FC < 0.67) than in non-responders.

#### 3.3.3 Column scatter analysis of differentially expressed EV membrane proteins

**Figure 3:**
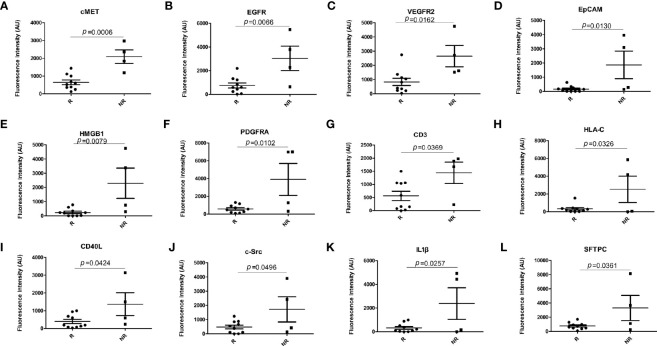
Bar graph analysis of differentially expressed EV membrane proteins at baseline between responders (n = 10) and non-responders (n = 4). **(A)** cMET; **(B)** EGFR; **(C)** VEGFR2; **(D)** EpCAM; **(E)** HMGB1; **(F)** PDGFRA; **(G)** CD3; **(H)** HLA-C; **(I)** CD40L; **(J)** c-Src; **(K)** IL-1β; **(L)** SFTPC. EV, extracellular vesicle; R, responders, NR, non-responders. *p*-values were calculated using the Mann–Whitney test.

A bar graph analysis was performed of the 12 significantly different EV membrane proteins in responders (3 PR and 7 SD) and non-responders (4 PD) at baseline. The results are shown in **Figures 3A–L**.

#### 3.3.4 Analysis of differentially expressed EV membrane proteins during treatment

**Figure 4:**
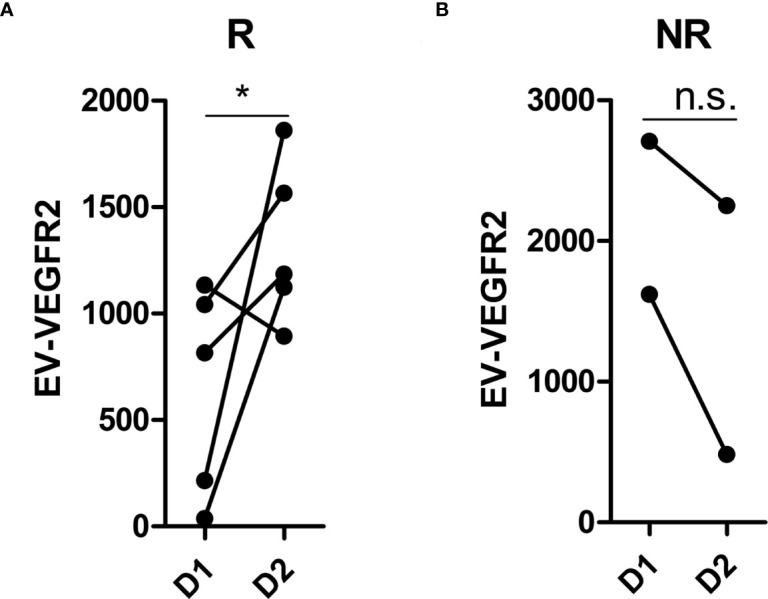
Analysis of differentially expressed EV membrane proteins during treatment. **(A)** the expression of VEGFR2 at D1 and D2 in responders (n = 5); **(B)** the expression of VEGFR2 at D1 and D2 in non-responders (n = 2). EV, extracellular vesicle; R, responders, NR, non-responders; D1, at baseline; D2, after treatment with immunotherapy combined with single-agent chemotherapy. * indicates *p* < 0.05. n.s. indicates no significant difference. *p*-values were calculated using the t test.

Plasma samples at stage D1 (baseline) and D2 (after treatment) were available in 7 patients who showed a PR (n = 2), SD (n = 3), or PD (n = 2) and were performed to identify significantly differentially expressed EV membrane proteins between stage D1 and D2. The results are shown in **Figure 4**. VEGFR2 expression was significantly increased at stage D2 in responders (**Figure 4A**) and decreased in non-responders (**Figure 4B**).

#### 3.3.5 Receiver operating characteristic curve analysis

**Figure 5:**
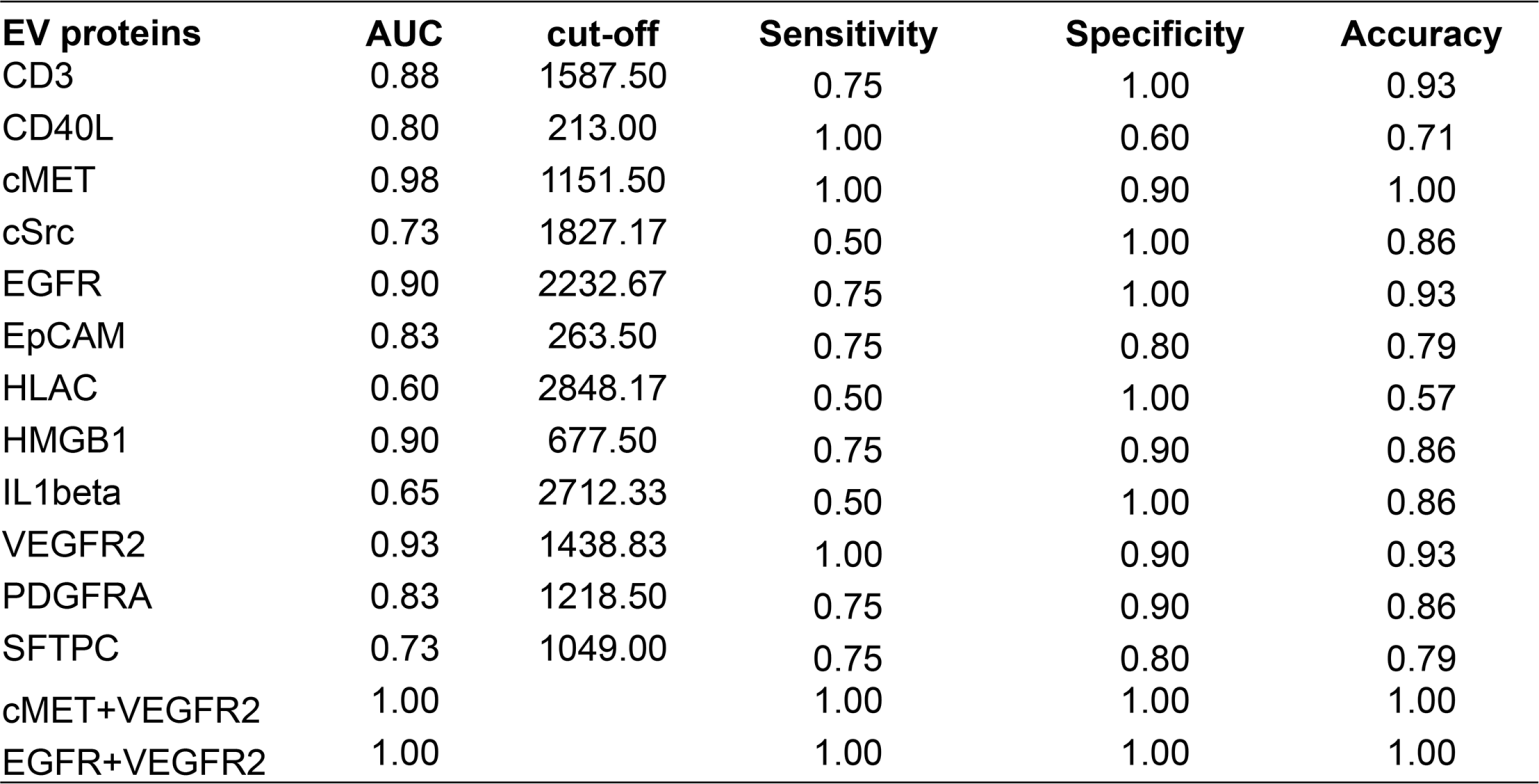
ROC curve analysis displaying the performance of EV proteins at baseline in distinguishing responders from non-responders. ROC, operating characteristic curve; EV, extracellular vesicle; AUC, area under the ROC curve.

**Figure 6:**
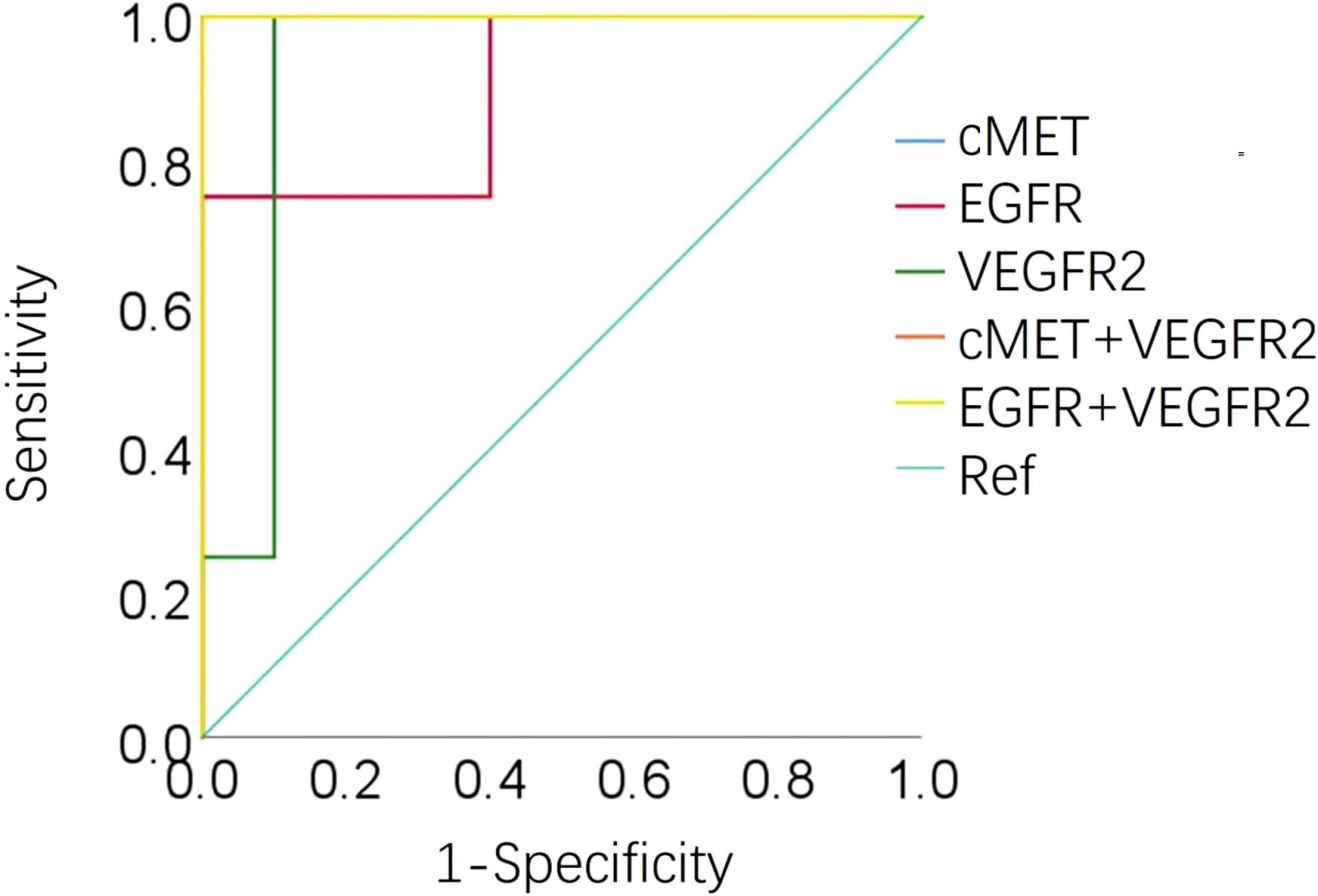
Area under the ROC curve of cMET, EGFR, VEGFR2, cEMT plus VEGFR2, and EGFR plus VEGFR2 at baseline in 14 patients. ROC, operating characteristic curve; AUC, area under the ROC curve; Ref, reference.

An AUC greater than 0.5 indicates that the diagnostic test has a certain diagnostic value. An AUC equal to 1 indicates a 100% accuracy of diagnostic tests. ROC curve analysis of the 12 EV membrane proteins at baseline negatively associated with immunotherapy combined with single-agent chemotherapy was performed to determine the diagnostic value of these proteins in identifying patients more likely to benefit from this treatment, including cMET. The results are shown in **Figures 5**, **6**. The AUC for cMET was 0.98 with a sensitivity of 100%, a specificity of 90%, and a diagnostic accuracy of 100%. Of note, the AUC was 1 with 100% sensitivity, 100% specificity, and 100% diagnostic accuracy when cMET was combined with VEGFR2 or EGFR was combined with VEGFR2.

#### 3.3.6 Kaplan–Meier survival analysis

**Figure 7:**
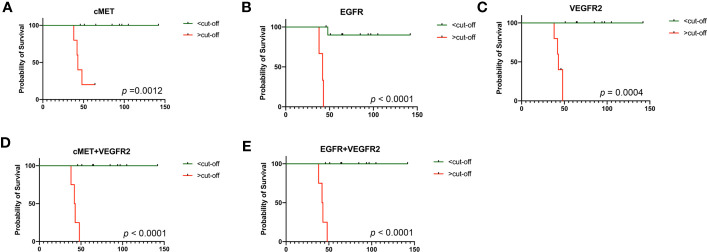
Kaplan–Meier (KM) analysis in 14 patients. **(A)** KM survival analysis by PFS in terms of cMET at baseline; **(B)** KM survival analysis by PFS in terms of EGFR at baseline; **(C)** KM survival analysis by PFS in terms of VEGFR2 at baseline; **(D)** KM survival analysis by PFS in terms of cMET combined with VEGFR2 at baseline; **(E)** KM survival analysis by PFS in terms of EGFR combined with VEGFR2 at baseline. PFS, progression-free survival. *p*-values were calculated using the log-rank test.

KM survival analyses of responders (3 PR and 7 SD) and non-responders (4 PD) were performed according to the threshold obtained by the ROC analyses. The results are shown in **Figure 7**. The PFS of patients with high expression of c-MET (**Figure 7A**, log-rank test, *p* = 0.0012), EGFR (**Figure 7B**, *p* < 0.0001), VEGFR2 (**Figure 7C**, log-rank test *p* = 0.0004), c-MET combined with VEGFR2 (**Figure 7D**, log-rank test, *p* < 0.0001), and EGFR combined with VEGFR2 (**Figure 7E**, log-rank test, *p* < 0.0001) at baseline was significantly shorter than that of those with low expression.

### 3.4 Adverse events

Grade 2 or higher adverse events were recorded in patients with metastatic NSCLC who received immunotherapy combined with single-agent chemotherapy. Hematologic toxicity (leucopenia, thrombocytopenia, and anemia) and gastrointestinal reactions (nausea and vomiting) occurred (**Table 3**). A total of 22 cases had immune-related adverse events, including 17 cases (56.7%) with thyroid dysfunction, 2 cases (6.7%) with cardiotoxicity, 1 case (3.3%) with nephrotoxicity, 1 case (3.3%) with pulmonary toxicity, and 1 case (3.3%) with skin rash. No deaths and discontinuation resulted from chemotherapy or immune-related adverse events.

**Table 3:** Adverse events.

Adverse events	Grade 2	Grade 3 or higher
**Leucopenia**	6 (20.0%)	3 (10.0%)
**Thrombocytopenia**	1 (3.3%)	0 (0%)
**Anemia**	1 (3.3%)	0 (0%)
**Gastrointestinal reaction**	9 (30.0%)	0 (0%)
**Skin rash**	0 (0%)	1 (3.3%)
**Neurotoxicity**	1 (3.3%)	0 (0%)
**Abnormal liver function**	1 (3.3%)	0 (0%)
**Total**	15 (50%)	4 (13.3%)

## 4 Discussion

Chemotherapy was historically believed to inhibit immunity; however, recent studies have indicated that some chemotherapeutic agents can induce DNA damage and promote an immune response. Immune checkpoint inhibitors represent a breakthrough in the first-line treatment of driver gene-negative advanced NSCLC. The KEYNOTE-189 (17) and KEYNOTE-407 (18) trials both showed that the median PFS (9.0 vs. 4.9 months and 8.0 vs. 5.1 months) and median OS (22.0 vs. 10.7 months and 17.1 vs. 11.6 months) of patients in the pembrolizumab combined with chemotherapy group were superior to those in the chemotherapy-alone group. In this work, the median PFS of patients who received immunotherapy plus single-agent chemotherapy was shorter than that reported in KEYNOTE-189 and KEYNOTE-407 trials (**Table S1**), which might be attributed to several factors. First, immunotherapy plus single-agent chemotherapy was adopted as a different treatment line in these studies, having been used as first-line setting in KEYNOTE-189 and KEYNOTE-407 trials and as second- or later-line in our work. Second, the same drugs were adopted for the chemotherapy plus pembrolizumab group in the KEYNOTE-189 (pembrolizumab+pemetrexed+platinum) and KEYNOTE-407 trials (pembrolizumab+carboplatin+paclitaxel/nab-paclitaxel), while different drugs were administered for patients who received immunotherapy (pembrolizumab/sintilimab/camrelizumab/nivolumab) and chemotherapy (albumin-bound paclitaxel/paclitaxel/vinorelbine/pemetrexed/gemcitabine). Third, our work had a relatively small sample size. Although the abovementioned details might result in the bias of our findings, metastatic NSCLC benefiting from immunotherapy plus chemotherapy with 76.7% DCR was observed in this study.

Second-line chemotherapy regimens for advanced NSCLC patients progressing from first-line therapy yield an ORR of only 10%, and monotherapy regimens with docetaxel and pemetrexed have achieved a median PFS of < 3 months. Immunotherapy has revolutionized the treatment of lung cancer. The CheckMate 078 study showed that nivolumab as a second-line therapy for NSCLC significantly prolonged OS (median: 12.0 vs. 9.6 months, *p* = 0.0006). The Chinese Society of Clinical Oncology (CSCO) guidelines recommend nivolumab (Class 1A evidence), docetaxel (Class 1A evidence), or pemetrexed (if not used as the first-line treatment) as the second-line treatment for advanced NSCLC. The KEYNOTE-010 (19) study showed that pembrolizumab significantly prolonged OS (median: 10.4 vs. 8.5 months, *p* = 0.0008), and the OAK (20) study showed that atezolizumab significantly prolonged OS compared with docetaxel (median: 13.8 vs. 9.6 months, *p* = 0.0003). These data show that advanced NSCLC patients treated with pembrolizumab or atezolizumab as a second-line treatment achieve good OS outcomes.

In this study, the ORR was 16.7%, the DCR was 76.7%, and the median PFS was 3.2 months in 30 patients with advanced NSCLC receiving immunotherapy combined with single-agent chemotherapy as a second- or later-line therapy. In prior studies, the ORR of docetaxel and pemetrexed monotherapy as second-line therapy was both <10%. The ORR of immunotherapy combined with single-agent chemotherapy achieved 16.7% in the current study.

These data indicate that the efficacy of immunotherapy combined with single-agent chemotherapy in this study was superior to that of the second-line standard of care based on the prior study (21). The median PFS of immunotherapy combined with single-agent chemotherapy was also superior to that of docetaxel and pemetrexed monotherapy as a second-line standard of care (3.2 vs. 2.6–3.0 months) (22). The 16.7% ORR of treatment in this study was comparable to that of second-line monotherapy nivolumab (16.6%) (7).

In addition, no serious adverse events were detected, and the treatment regimen was well tolerated. Taken together, the data indicate that immunotherapy combined with single-agent chemotherapy as a second- or later-line therapy is effective against advanced NSCLC, improving patient survival with a good safety profile. Although some grade 2 or higher adverse events occurred, these adverse events were relieved with the discontinuation of the treatment.

EVs transfer nucleic acids and proteins in the cell–cell communication involved in a variety of biological processes (23–25). In addition to their enrichment in nucleic acids, EVs are also a specific composition of membrane proteins (26). The sandwich immunoassay-based EV Array was recently developed for EV membrane protein determination (27). In comparison to conventional protein detection methods, the EV Array can detect EVs expression in a high-throughput manner. Moreover, this platform is highly sensitive with a small amount of protein samples under the unpurified condition. EVs can protect cargo from degradation and indicate the tissue of origin. The data analysis showed that EV membrane proteins, such as c-MET, EGFR, VEGFR2, CD3, CD40 ligand (CD40L), SRC proto-oncogene non-receptor tyrosine kinase (C-Src), epithelial cell adhesion molecule (EpCAM), major histocompatibility complex, class I C (HLAC), high-mobility group box 1 (HMGB1), interleukin 1 beta (IL1beta), platelet-derived growth factor receptor alpha (PDGFRA), and surfactant protein C (SFTPC), were at high levels in the plasma of patients who did not respond to the treatment and at low levels in the plasma of those who showed a clinical benefit. Therefore, the levels of these 12 EV membrane proteins are inversely associated with the efficacy of immunotherapy combined with single-agent chemotherapy for lung cancer. ROC and KM curve analyses showed that PFS was significantly shorter in patients with high levels of c-MET, EGFR, and VEGFR2 than in those with low levels, suggesting that patients with low levels of these proteins have a better prognosis. These data indicate that EVs related to treatment efficacy have potential value and might be used as markers in identifying patients who may not respond to treatment to guide clinical decision-making. During treatment, the expression of the EV membrane protein VEGFR2 was significantly increased in the responder group and decreased in the non-responder group, indicating that the change in VEGFR2 expression on the EV membrane can be used as a marker to monitor drug efficacy.

This study has some limitations. First, the sample size of this study was small; thus, large prospective cohort studies are warranted to verify the predictive values of EV markers. Second, this was a single-arm, single-center study without a control group, and bias could not be avoided. Further large prospective cohort studies are warranted to investigate the efficacy of immunotherapy plus single-agent chemotherapy vs. platinum-containing chemotherapy/immunotherapy alone as a second- or later-line therapy for metastatic non-small cell lung cancer. Third, despite our findings of the cut-offs of the EV proteins in identifying patients who would more likely benefit from immunotherapy combined with single-agent chemotherapy, the clinical evidence of the cut-offs of the EV proteins should be verified in a large, multi-center, randomized clinical trial in further work. Forth, due to the small number of patients participating in the study, the efficacy of immunotherapy plus single-agent chemotherapy as a second- or later-line therapy for metastatic non-small cell lung cancer was not explored in terms of actionable alteration status and PD-L1 status. Further work is needed to investigate its efficacy in patients with vs. without actionable alterations and high vs. low PD-L1 expression.

This study thoroughly assessed the efficacy and safety of immunotherapy combined with single-agent chemotherapy. The results could be used to inform clinical practice. It also explored the clinical value of using EV surface membrane protein expression to predict and monitor the efficacy of immunotherapy combined with single-agent chemotherapy. Thus, this study may facilitate treatment selection and decision-making, improve our understanding of microenvironmental changes associated with immunotherapy, and lead to the improvement of the currently available treatment options.

## Data availability statement

The original contributions presented in the study are included in the article/**Supplementary Material**. Further inquiries can be directed to the corresponding authors.

## Ethics statement

The studies involving humans were approved by the Ethics Committee of Cancer Hospital of Chinese Academy of Medical Sciences. The studies were conducted in accordance with the local legislation and institutional requirements. The participants provided their written informed consent to participate in this study.

## Author contributions

Conception and design: YL. Data acquisition: DC, YL, LL, MW, XH, and JJ. Data analysis: DC, YL, WL, LY, and MF. Data interpretation: YL, DC, and YS. Drafting the manuscript: DC. Critical revision of the manuscript: YL. All authors contributed to the article and approved the submitted version.

## Supplementary material

Supplementary Figure 1 The KM curve for PFS of 30 eligible patients. KM, Kaplan–Meier curves; PFS, progression-free survival.
