# Supplementary material for: Efficacy and safety of immunotherapy combined with single-agent chemotherapy as second- or later-line therapy for metastatic non-small cell lung cancer
Source: Front Immunol. 2023 Sep 18;14:1086479. doi: 10.3389/fimmu.2023.1086479 (PMC10547148; doi:10.3389/fimmu.2023.1086479)
Supplement: Supplementary file 1 [file Table_1.docx]

**Table S1 The treatment of patients**

| **Treatment** | **Number of patients (%)** |
| --- | --- |
| **Immunotherapy (n = 30)** | |
| Pembrolizumab | 14 (46.7%) |
| Sintilimab | 11 (36.7%) |
| Camrelizumab | 3 (10.0%) |
| Nivolumab | 2 (6.6%) |
| **Single-agent chemotherapy (n = 30)** | |
| Albumin-bound paclitaxel | 15 (50.0%) |
| Paclitaxel | 6 (20.0%) |
| Vinorelbine | 5 (16.7%) |
| Pemetrexed | 3 (10.0%) |
| Gemcitabine | 1 (3.3%) |
