# Supplementary material for: Efficacy and safety of immunotherapy combined with single-agent chemotherapy as second- or later-line therapy for metastatic non-small cell lung cancer
Source: Front Immunol. 2023 Sep 18;14:1086479. doi: 10.3389/fimmu.2023.1086479 (PMC10547148; doi:10.3389/fimmu.2023.1086479)
Supplement: Supplementary file 2 [file Table_2.docx]

**Table S2. The 57 proteins for EV expression array**

| PDL1 | PDL2 | CTLA-4 | CD73 | HER2 | HER3 | TGF-β | HSP70 | ICOS | IDO1 |
| --- | --- | --- | --- | --- | --- | --- | --- | --- | --- |
| CD4 | CD8 | ARG1 | HIF1A | CD39 | CD39 | CD86 | CD80 | CD3 | TROP2 |
| IGF-1R | CD64 | Mesothelin | ICAM1 | LAG-3 | IL-15R | CD40L | HLA-A | HLA-B | HLA-C |
| HLA-DQ | HLA-DR | HLA-DP | HMGB1 | CD47 | CD24 | CD19 | ANXA6 | CXCL13 | c-Kit |
| c-Met | VEGFR2 | PDGFR | VEGFR3 | c-Src | VEGFR1 | FGFR | EGFR | INF-gamma | IL6 |
| MUC1 | THBS1/2 | EpCAM | SFTPC | IL-1β | CD63 | CD81 | CD9 |  |  |

EV, extracellular vesicle.
