# Supplementary material for: Efficacy and safety of immunotherapy combined with single-agent chemotherapy as second- or later-line therapy for metastatic non-small cell lung cancer
Source: Front Immunol. 2023 Sep 18;14:1086479. doi: 10.3389/fimmu.2023.1086479 (PMC10547148; doi:10.3389/fimmu.2023.1086479)
Supplement: Supplementary file 3 [file Table_3.docx]

**Table S3 The details of KEYNOTE-189/407 trails and our study**

| **Publication** | **Year** | **Patients** | **No. of patients** | **Treatment regimen** | **Treatment line** | **Median PFS (mo)** | **Median OS (mo)** |
| --- | --- | --- | --- | --- | --- | --- | --- |
| KEYNOTE-189 | 2020 | Metastatic nonsquamous NSCLC | 410 vs. 206 | Pembro+pemetrexed+platinum plus vs.  placebo+pemetrexed+platinum | First-line | 9.0 vs. 4.9 | 22.0 vs. 10.7 |
| KEYNOTE-407 | 2020 | Metastatic squamous NSCLC | 278 vs. 281 | Pembro+carboplatin+paclitaxel/nab-paclitaxel vs. placebo+carboplatin+paclitaxel/nab-paclitaxel | First-line | 8.0 vs. 5.1 | 17.1 vs. 11.6 |
| Our work |  | Metastatic NSCLC | 30 | Pembrolizumab/sintilimab/camrelizumab/nivolumab+albumin-bound paclitaxel/paclitaxel/vinorelbine  /pemetrexed/gemcitabine | Second- or later-line | 3.2 | NA |

NSCLC, non-small cell lung cancer; No., number; pembro, pembrolizumab; chemo, chemotherapy; PFS, progression-free survival; OS, overall survival; mo, month; NA, not applicable.
