# Supplementary figures and images for: Efficacy and safety of immunotherapy combined with single-agent chemotherapy as second- or later-line therapy for metastatic non-small cell lung cancer
Source: Front Immunol. 2023 Sep 18;14:1086479. doi: 10.3389/fimmu.2023.1086479 (PMC10547148; doi:10.3389/fimmu.2023.1086479)

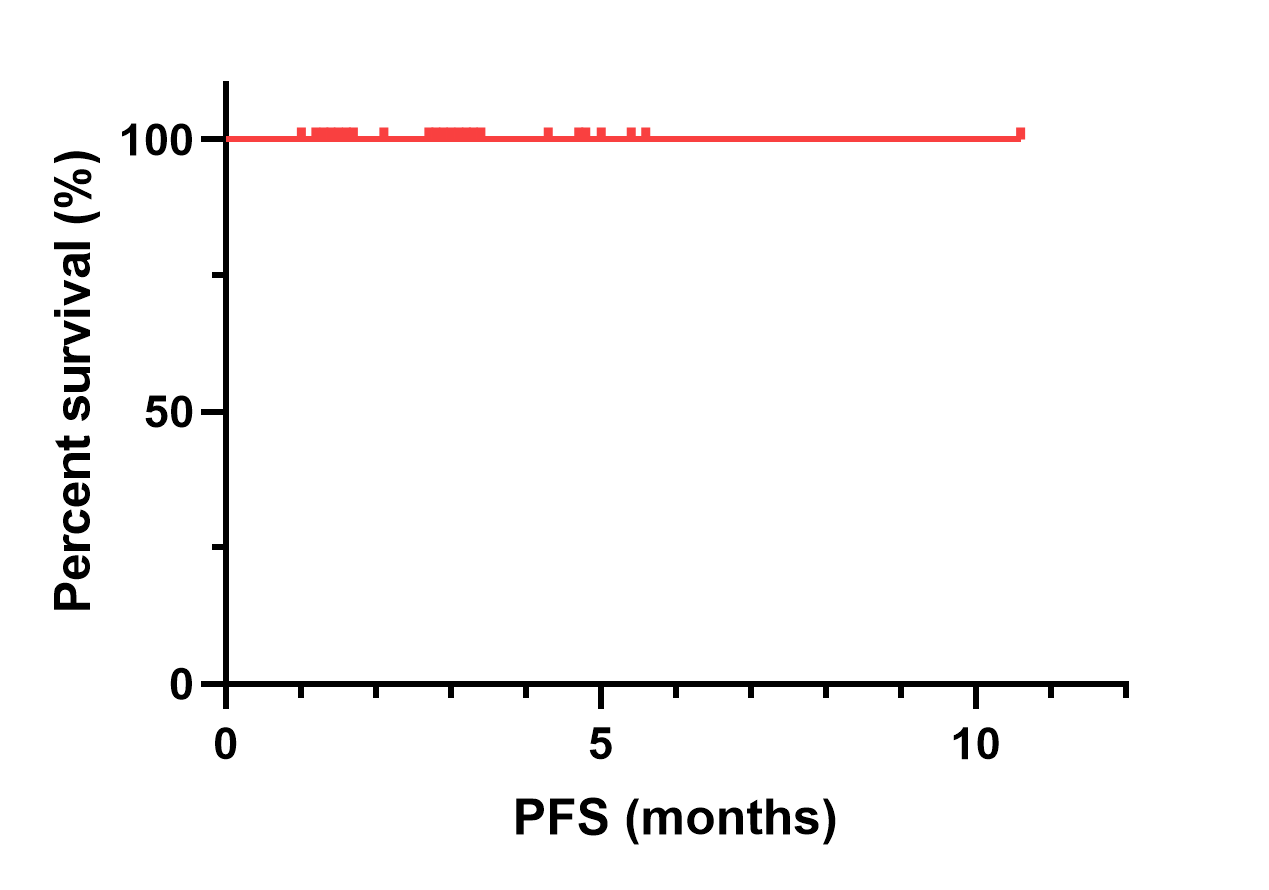

Supplement: Supplementary Figure 1 — The KM curve for PFS of 30 eligible patients. KM, Kaplan–Meier curves; PFS, progression-free survival. [file Image_1.tif]
